# Tongue laceration in a patient taking antiplatelet agents during transcranial motor-evoked potential monitoring: a case report

**DOI:** 10.1186/s40981-022-00593-6

**Published:** 2022-12-29

**Authors:** Katsuhiro Matsumoto, Hideyuki Nakagawa, Akira Kitamura

**Affiliations:** Department of Anesthesiology, Saitama Medical University International Medical Center, 1397-1 Yamane, Hidaka-shi, Saitama, 350-1298 Japan

**Keywords:** Motor-evoked potential, Tongue bite injury, Carotid endarterectomy, Antiplatelet agent

## Abstract

**Background:**

Transcranial motor-evoked potential (Tc-MEP) monitoring is usually performed during surgeries involving a risk of damaging brain motor areas. However, it involves a risk of bite injuries. We report a case of severe tongue laceration from Tc-MEP stimulation during carotid endarterectomy (CEA) in a patient taking antiplatelet agents.

**Case presentation:**

A 74-year-old man on antiplatelet therapy was scheduled for CEA under general anesthesia with intraoperative Tc-MEP monitoring. Bite blocks were not inserted. Postoperatively, we observed a tongue laceration with severe bleeding, which was sutured. The difficulties in tongue movement persisted for ≥ 1 month postoperatively.

**Conclusions:**

Bite injuries during Tc-MEP may induce severe bleeding in patients on antiplatelets. The complications of tongue bite injuries may persist, decreasing the patients’ quality of life. Hence, during Tc-MEP monitoring, it is important to use soft bite blocks and to check the patient’s face and the position of the tracheal tube intraoperatively.

## Background

Transcranial motor-evoked potential (Tc-MEP) monitoring is widely used to evaluate the function of motor pathways intraoperatively. However, transcranial electrical stimulation causes masseter muscle contraction, resulting in bite-induced damage to the tongue, teeth, and endotracheal tubes [1]. Furthermore, most patients undergoing carotid endarterectomy (CEA) take antithrombotic medication for the primary or secondary prevention of cerebrovascular, peripheral vascular, and coronary artery diseases [2]. In this report, we describe a case of a male patient taking antiplatelet agents whose tongue was injured severely by Tc-MEP stimulation during CEA.

## Case presentation

The patient was a 74-year-old man (American Society of Anesthesiologists physical status 2) with hypertension and a history of asymptomatic cerebral infarction. He was scheduled for right-sided CEA. Preoperative laboratory tests revealed no coagulation abnormalities. The patient was taking clopidogrel 75 mg/day and cilostazol 200 mg/day, with the latter being discontinued 3 days before surgery. The patient had no history of ingestion or swallowing dysfunction.

In the operating room, electrocardiograms, noninvasive blood pressure, invasive radial artery pressure, percutaneous oxygen saturation, end-tidal carbon dioxide concentration, and rectal temperature were monitored. General anesthesia was induced using propofol, fentanyl, and rocuronium. An endotracheal tube (spiral tube, internal diameter: 8.0 mm) was placed and fixed at the left corner of the mouth. There were no post-intubation abnormalities in the oral mucosa or teeth. Bite blocks were not inserted. Anesthesia was maintained using propofol at target concentration of 2.0–3.0 μg/ml and remifentanil 0.3–0.4 μg/kg/min. Rocuronium was not administered again after anesthesia induction. The patient was placed in the supine position; subsequently, his neck was rotated and flexed approximately 40° to the left to secure the operative field. Before surgery, the patient’s activated clotting time (ACT) was 185 s, and 2000 units of heparin were administered. Intraoperatively, 6000 units of heparin were administered, and the ACT was maintained at 218–281 s.

Intraoperative neurophysiological monitoring was performed using MEP and somatosensory-evoked potential. MEP was evoked through transcranial pulse-train stimulation at a constant voltage (350 V) using a multimodal neuromonitor (MEE-2000 Neuromaster G1; NIHON KOHDEN, Tokyo, Japan). Short trains of five electrical pulses (frequency: 500 Hz, duration of each stimulus: 0.05 ms) were applied through electrodes to C3 and C4 (international 10–20 electroencephalography system). Compound muscle action potentials were recorded in the left abductor pollicis brevis, brachioradialis, gastrocnemius, and anterior tibial muscles.

**Fig. 1:**
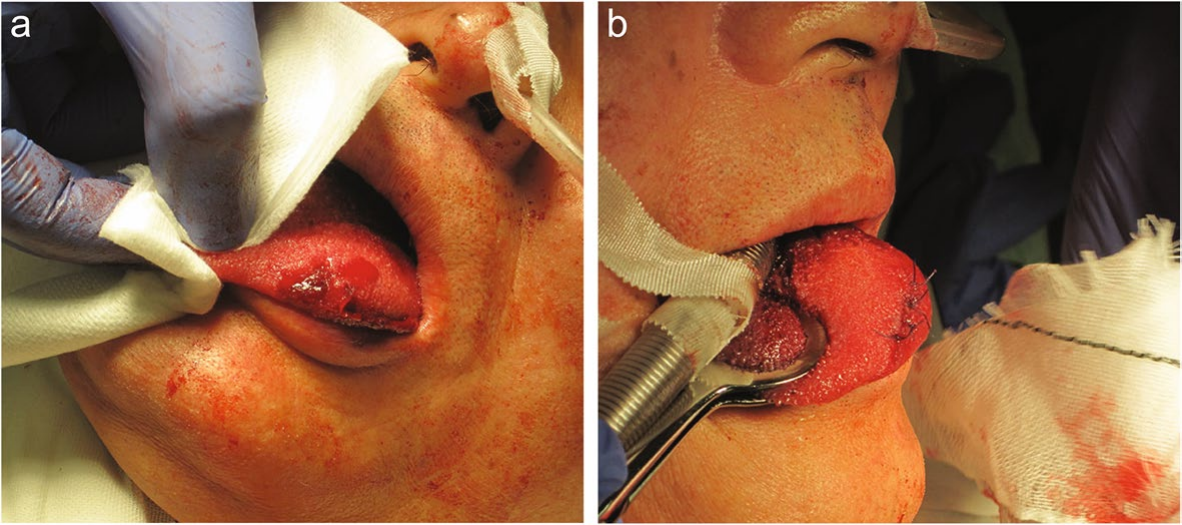
Photograph of the patient’s tongue. This photograph shows the tongue bite injury on the left side (**a**) and the sutured laceration (**b**)

After the surgery, we observed a hematoma and laceration (10 mm in length, 5 mm in depth) on the left anterior dorsum of the tongue (Fig. 1a). The wire-reinforced endotracheal tube was not damaged. Compression with gauze did not achieve hemostasis; hence, otolaryngologic consultation was sought, and the injury was sutured (six stitches, 3–0 vicryl) (Fig. 1b). After confirming hemostasis, no new bleeding or swelling was observed for approximately 1 h. Therefore, the patient was extubated. The patient could communicate without respiratory discomfort post-extubation. His ACT was 188 s, and heparinization was not reversed using protamine. The surgery duration was 3 h and 49 min, the anesthesia duration was 6 h and 46 min, and the volume of blood loss during surgery was 23 ml. The patient was transferred to the intensive care unit in stable condition.

On the first postoperative day, the patient complained of tongue discomfort, dysphagia, and dysarthria. Therefore, the patient was placed on a porridge diet. Examination with the tongue protruded revealed no tongue deviation or swelling. Hoarseness was not observed, and laryngoscopy revealed no abnormalities other than slight swelling of the right arytenoid cartilage. The tongue pain gradually resolved, but the difficulty in tongue movement persisted. On the 11th postoperative day, the patient could consume a regular diet. He showed no signs of postoperative dysgeusia. One month after discharge, the scar on his tongue had disappeared; however, the difficulties in tongue movement persisted. Three months after discharge, the patient had no difficulties in swallowing or speaking.

## Discussion

This article presents a case of severe tongue injury caused by Tc-MEP stimulation during CEA. In this case, hemostasis was difficult to achieve since the patient was on antiplatelet therapy. He required suture repair for the tongue laceration, and the patient had difficulty moving his tongue for ≥ 1 month postoperatively.

The reported incidence of bite injuries related to Tc-MEP ranges from 0.2 to 6.5% [1, 3, 4]. A survey conducted by the Japanese Society of Anesthesiologists in 2018 showed that bite injuries are reported by 3–4% of its member institutions [5]. Bite injuries are relatively common complications of Tc-MEP in our country. Although one study reported that 25 of 109 bite injuries (23%) required suturing [3], few lacerations require otolaryngologic consultation. In our case, hemostasis could not be achieved through compression with gauze; therefore, suturing was required. Before the surgery, the patient was on antiplatelet therapy. Preoperative clopidogrel treatment is associated with an increased risk of neck bleeding after CEA [2]. Given the difficulty in keeping the tongue still due to constant movement and stimulation while eating and speaking, there is a high risk of postoperative rebleeding. Therefore, we considered suture hemostasis necessary, and as a result, postoperative rebleeding was prevented.

Heparinization might have also contributed to this bleeding since heparin was administered during CEA. However, we thought the involvement of heparin in this bleeding was minimal and did not use protamine for the following reasons: first, it had been more than 100 min (more than the half-life of heparin) since the last heparin administration, and the final ACT had returned to its pre-heparinization value; second, protamine administration may cause serious anaphylactic reactions [6]; and third, local hemostasis by suturing was possible. Due to these reasons, we decided not to neutralize heparin with protamine.

In patients with severe tongue hematoma, there is an additional risk of airway obstruction after tracheal extubation [3]. In our case, the wounds were observed for approximately 1 h after suture hemostasis. Given the lack of tongue swelling and the low risk of airway complications, the patient was extubated.

The patient complained of difficulty in tongue movement for ≥ 1 month postoperatively. A study on bite injuries in the oral mucosa caused by Tc-MEP found that almost all patients recovered by the 12th postoperative day [4]. Hypoglossal nerve injury may occur due to CEA manipulation [7]. The nerve injury can cause dysarthria, dysphagia, and tongue deviation to the affected side. Most of these symptoms persist for ≥ 1 month. In our case, postoperative evaluation did not show tongue deviation. This indicates that there was no hypoglossal nerve damage due to CEA manipulation, and that the difficulty in tongue movement persisting for ≥ 1 month was caused by the bite injury alone.

The three factors that contributed to the tongue laceration in our case were (1) the high stimulus intensity of Tc-MEP, (2) the lack of bite blocks, and (3) not observing the patient’s face periodically during surgery. High-voltage transcranial electrical stimulation can induce masseter muscle contractions and may increase the risk of bite injuries [1, 3]. In our case, the stimulus intensity of Tc-MEP was 350 V, which was considered high. Although high intensity of stimulation has been considered as a risk factor of bite injury during Tc-MEP monitoring, we were unable to get sufficient Tc-MEP responses at less than 350 V. We thought this high stimulus intensity was one of the causes of tongue laceration. In addition, the patient’s tongue moved to the left side and slipped between the upper and lower teeth because his head tilted to the left, leading to the bite injury. In the guidelines, the use of soft dental blocks and rolled-up gauze is recommended to prevent the tongue from protruding and the teeth from closing on the tongue [5]. Moreover, frequent intraoperative observation of the patient’s face, the tracheal tube, and the bite blocks is also suggested.

In conclusion, we encountered a case of severe tongue laceration during Tc-MEP in a patient taking antiplatelet agents whose tongue discomfort persisted for a long time. The novelty of this case report may be limited because many tongue lacerations caused by Tc-MEP have been reported previously. However, we think that our case has educational value not only to remind the anesthesiologists of the importance of using soft bite blocks and checking the patient’s face and the tracheal tube periodically during CEA surgery but also to warn the possibility of this severe complication caused by Tc-MEP.

## Data Availability

Not applicable

## Abbreviations

Tc-MEP: Transcranial motor-evoked potential
CEA: Carotid endarterectomy
ACT: Activated clotting time
